# A Comparative Analysis of Resuscitative Endovascular Balloon Occlusion of the Aorta (REBOA), Resuscitative Thoracotomy, and Nonprocedural Care for the Management of Life-Threatening Traumatic Torso Hemorrhage

**DOI:** 10.7759/cureus.80210

**Published:** 2025-03-07

**Authors:** Samantha Spence, Jennifer Fox, Thomas P Hoag, Madison Yeager, Claire Schroll, Thomas Koch, William Rudder, Matthew Whalen, Dana Alshekhlee, Blake Miyamoto, Diana Fan, Christopher Sandoval, Raymond I Okeke, John Culhane

**Affiliations:** 1 Department of Trauma Surgery, Saint Louis University School of Medicine, St. Louis, USA; 2 Department of Surgery/Emergency Medicine, Sisters of St. Mary (SSM) Health Saint Louis University Hospital, St. Louis, USA; 3 Department of General Surgery, Sisters of St. Mary (SSM) Health Saint Louis University Hospital, St. Louis, USA; 4 Department of Trauma Surgery, Sisters of St. Mary (SSM) Health Saint Louis University Hospital, St. Louis, USA

**Keywords:** aortic occlusion, emergency department, noncompressible torso hemorrhage, reboa, resuscitation, thoracotomy, trauma

## Abstract

Objective: This study aimed to compare the mortality of resuscitative endovascular balloon occlusion of the aorta (REBOA) with emergency department thoracotomy (EDT) and nonprocedural resuscitation (NPR) in the initial resuscitation of life-threatening acute traumatic hemorrhage.

Methods: We performed a retrospective chart review of all patients who presented at a single urban level I trauma center with noncompressible torso hemorrhage between January 1, 2012, and October 31, 2022. Patients with a life-threatening injury (Abbreviated Injury Scale score >3) involving significant bleeding in the thorax or abdomen were included. Exclusion criteria were life-threatening head injury and mechanism of ground-level fall. Patients were classified according to the primary means of resuscitation: EDT, REBOA, or blood products and fluid (hemostatic resuscitation) without procedural intervention. The primary outcome was in-hospital mortality. Univariate and multivariate analyses were performed. Covariates included patient demographics, mechanism of injury, and injury severity.

Results: Two hundred sixty-seven cases met the criteria for inclusion. Initial resuscitation was EDT for 71 patients, REBOA for 17, and hemostatic resuscitation only for 179. Mortality rates for EDT compared to REBOA were 64 (90.1%) vs. 10 (58.8%) (p = 0.0051). The adjusted odds ratio (OR) was 0.09 (p = 0.009). The mortality rate for hemostatic resuscitation alone was 41 (22.9%) (p < 0.001) with an OR of 0.02 compared with those who received either EDT or REBOA. This finding remained significant when REBOA and conservative management were compared directly, excluding EDT patients (p = 0.0033).

Conclusions: Mortality associated with EDT is higher than that of REBOA. Mortality for both is higher than nonprocedural hemostatic resuscitation. REBOA is an appropriate salvage maneuver for patients with life-threatening abdominopelvic hemorrhage. However, our results do not support liberalizing its indications to patients responsive to hemostatic resuscitation.

## Introduction

Uncontrollable hemorrhage is a major cause of mortality in trauma, accounting for nearly 40% of deaths in the civilian setting. In fact, trauma is currently the leading cause of death in the United States for patients between the ages of 1 and 46. Furthermore, hemorrhage accounts for the vast majority of preventable deaths, defined as casualties that could have been avoided with timely and appropriate intervention [1]. Hemorrhage can further be classified into compressible or incompressible categories. Noncompressible torso hemorrhage (NCTH) refers to a severe bleeding injury in the chest or abdomen, often involving major blood vessels or organs, where direct pressure cannot be applied to control the blood loss. This often leads to a life-threatening situation that requires immediate surgical intervention to control the bleeding [2]. There has been an appreciable reduction in mortality due to compressible hemorrhage with the use of tourniquets and topical hemostats [1]. However, the treatment for NCTH remains a significant clinical challenge as it is often hindered by difficulty accessing the source of bleeding and delays in its detection. As such, NCTH has been found to have a mortality rate of 67% following injuries deemed to be otherwise potentially survivable [3]. These patients are often managed with aggressive fluid resuscitation and aortic occlusion when necessary.

The goal of aortic occlusion in the setting of exsanguinating torso hemorrhage is to obtain temporary hemodynamic stability, maintaining perfusion to the brain and allowing time for definitive hemorrhage control. Emergency department thoracotomy (EDT) is the traditional salvage maneuver for trauma patients in full or impending cardiac arrest. It is most widely accepted for blunt-trauma patients with a cardiopulmonary resuscitation (CPR) duration of less than 10 minutes, penetrating-trauma patients with a CPR duration of less than 15 minutes, and patients who have penetrating trauma to the chest accompanied by asystole and evidence of pericardial tamponade [4,5]. It has also been extended to include the management of massive hemoperitoneum to prevent cardiac decompensation before laparotomy [6]. This procedure involves making a left anterolateral thoracotomy incision in the fourth or fifth intercostal space by penetrating and separating the intercostal muscles and the underlying pleura. This incision is subsequently extended anteriorly toward the sternum and posteriorly towards the mid-axillary line. Rib spreaders are then introduced between the ribs and expanded maximally to optimize exposure. After entering the chest, the aorta is cross-clamped above the diaphragm while open cardiac massage and internal defibrillation can be performed as needed [7]. This temporizing approach is intended to redirect circulation temporarily and deliver the patient to the operating room for definitive repair. However, given the inherent morbidity of this maneuver and survival rate of only 7.4%-8.5% [7], physicians have started to look toward less invasive means of aortic occlusion.

Resuscitative endovascular balloon occlusion of the aorta (REBOA) is an intra-aortic balloon occlusion device introduced into the descending aorta percutaneously via femoral access [8]. Over the past decade, REBOA has emerged as a promising technique to control noncompressible traumatic torso hemorrhage [6]. This procedure has been shown to improve cerebral and coronary blood flow during CPR [9-11] and yields a positive effect on systolic blood pressure [12] with less physiologic disturbance. With the increased use of REBOA, treatment guidelines from the American College of Surgeons Committee were published, recommending REBOA for the treatment of life-threatening hemorrhage below the diaphragm. Occlusion of the distal thoracic aorta (Zone 1) or distal abdominal aorta (Zone 3) allows for the management of intra-abdominal or pelvic and more distal hemorrhage, respectively [13]. REBOA is indicated in selected adult patients with cardiac arrest (<10 minutes) secondary to exsanguination from subdiaphragmatic hemorrhage, with severe hypovolemic shock and systemic blood pressure <70 mmHg, or with nonresponsiveness/partial responsiveness to rapid volume resuscitation with suspected or diagnosed intra-abdominal hemorrhage [14]. REBOA is currently contraindicated for hemorrhage, including the major thoracic vessels, and, as such, no treatment guidelines exist for the use of REBOA in the management of chest injuries [13]. The procedure can be performed in trauma resuscitation areas or operating rooms by a specially trained surgeon and involves the insertion of a guidewire into a femoral or brachial arterial line followed by sequential dilation. The balloon catheter is then inserted over the guidewire to the appropriate depth, and the balloon is inflated with saline/contrast to achieve full or partial occlusion of the aorta, temporarily controlling NCTH and improving hemodynamics until definitive surgical or endovascular hemorrhage control can be achieved [14]. Despite the less invasive nature of REBOA and the potential for faster aortic occlusion, studies remain limited and heterogeneous regarding the safety, efficacy, and survival rates of REBOA compared to EDT [15,16]. This study compares the survival rates of patients treated with REBOA vs. EDT and examines both procedures against nonprocedural resuscitation (NPR) for managing hemorrhagic trauma. Our hypothesis is that REBOA offers survival benefits compared to EDT and nonprocedural initial resuscitation.

## Materials and methods

Data source

This is a retrospective cohort study analyzing patients who underwent REBOA, EDT, or nonprocedural volume resuscitation at a single urban level I trauma center. Data were retrieved from a prospectively maintained hospital Trauma Registry, including but not limited to patient demographics, injury details, emergency medical service data, patient vitals, procedure data, and hospital admission and discharge data. This process was supplemented by direct chart review as needed. We reviewed all trauma patients who presented between January 1, 2012, and October 31, 2022. Only patients with injuries resulting in significant internal bleeding were considered. Patients were included if they had an Abbreviated Injury Scale (AIS) score >3 indicating a life-threatening injury with an associated AIS body region of 4 or 5 corresponding to the thorax and abdomen/pelvis, respectively. For polytrauma patients, we used their most severe injury, represented by the highest reported AIS score. Patients with AIS scores of 6 were not included, as this represents a nonsurvivable injury. Patients were excluded if their injury resulted from a ground level fall, as the greatest cause of morbidity is almost always head trauma, or if they did not present with major hemorrhage.

Measures

Demographic characteristics collected were age, sex, and race. The mechanism of injury (MOI) was categorized into gunshot wound (GSW), penetrating stab wound, motor vehicle collision (MVC), pedestrian vs. motor vehicle collision, motorcycle collision, and other. Injury Severity Score (ISS) and Trauma and Injury Severity Score (TRISS) were calculated for each patient. The ISS standardizes the severity of traumatic injury based on the worst injury of six body systems. TRISS is reported as a percent chance of survival and considers the ISS and revised trauma score, a physiologic index that estimates preinterventional mortality risk. The type of intervention, classified as EDT, REBOA, or NPR, was documented for each patient. Of note, all hemorrhaging patients receive fluid and blood product resuscitation as a standard of care, regardless of the indication for surgical intervention. Thus, the NPR group was considered the control group. The choice of procedure was based on the surgeon's judgment and clinical gestalt within their accepted indications. NPR was defined as resuscitation with blood products and isotonic fluids as needed to address hemodynamic instability without surgical intervention. The primary outcome was in-hospital mortality.

Statistical analysis

Baseline characteristics were summarized by calculating the mean and standard deviation for continuous variables, while frequencies with percentages were used for categorical variables. These characteristics were stratified according to the procedure type (REBOA, EDT, and NPR). A univariate analysis was performed using chi-square for mortality as a categorical variable. Multivariate logistic regression was applied for multivariate analysis. Covariates were ISS, TRISS, age at presentation, sex, cardiac arrest status, and MOI. A p value of <0.05 was considered to be significant. The primary independent variable was procedure type, with EDT as the reference group. The dependent variable of interest was in-hospital mortality.

## Results

Of the 696 patients screened, 267 met the inclusion criteria. Seventy-one patients underwent EDT, 17 were treated with femoral-access REBOA, and 179 were managed with volume resuscitation alone.

Baseline characteristics

The patient population consisted mainly of young to middle-aged male individuals across all three treatment groups. GSWs represented the most common MOI in the groups receiving procedural intervention, whereas MVC was most common in the NPR group. Other mechanisms of injury included assault, boating accident, and nonground level fall, all of which were considered blunt. ISS was well-balanced across the three treatment groups. TRISS was notably lower in the EDT group. A total of 43.7% of EDT patients arrived at the emergency department (ED) with cardiac arrest compared to only 3.9% of NPR patients. These differences reveal some baseline imbalance in patient acuity (Table 1).

**Table 1:** Comparison of mortality rates in patients receiving REBOA, EDT, and NPR Chi-square results show a significantly higher mortality rate associated with EDT and with either procedural intervention when compared to NPR (p < 0.001) EDT: emergency department thoracotomy; REBOA: resuscitative endovascular balloon occlusion of the aorta; NPR: nonprocedural resuscitation

Procedure	Death		Total (n = 267)
	No (n = 152)	Yes (n = 115)	
EDT	7 (0.099)	64 (0.901)	71 (0.266)
REBOA	7 (0.412)	10 (0.588)	17 (0.064)
NPR	138 (0.771)	41 (0.229)	179 (0.670)

Univariate analysis

**Figure 1:**
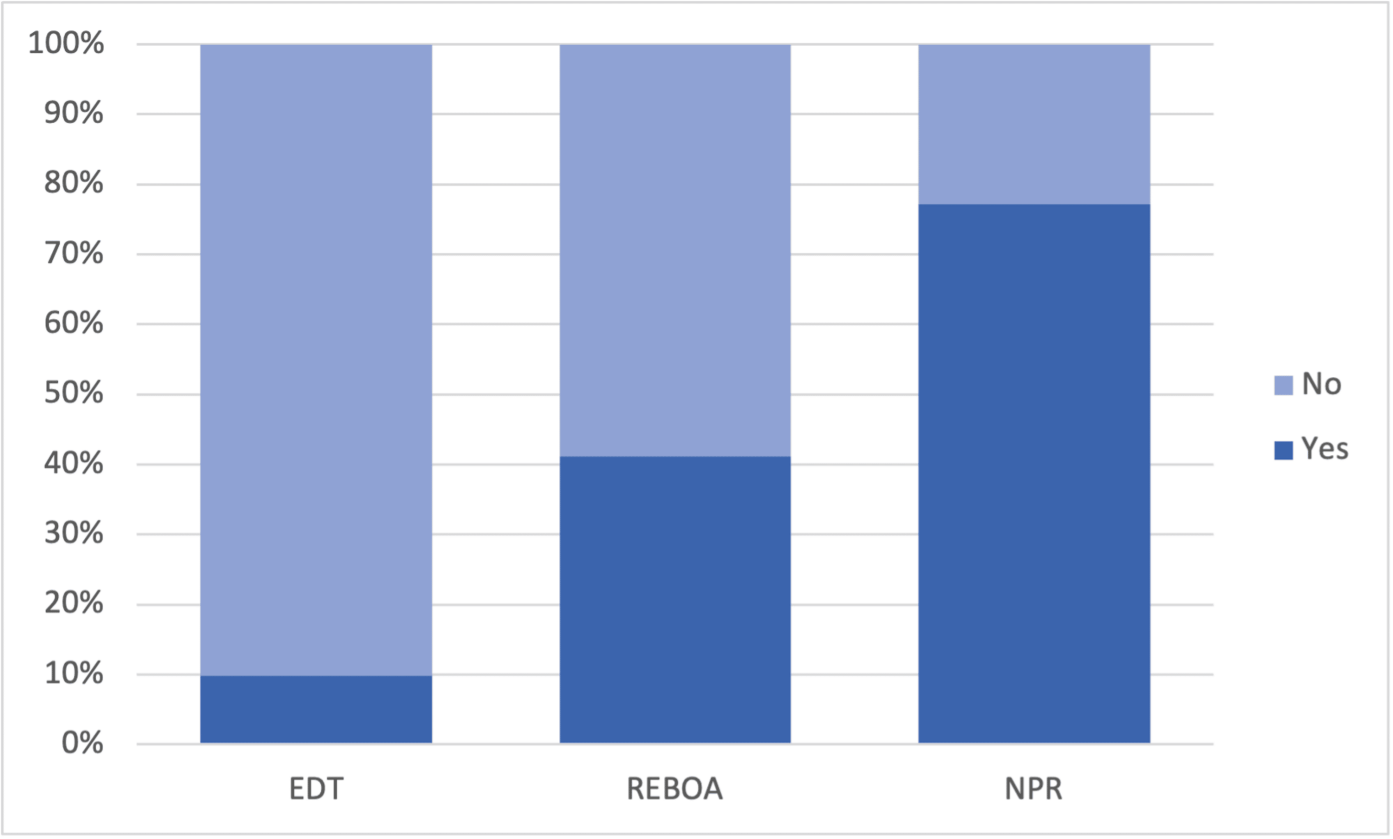
Survival rate comparison among patients receiving REBOA, EDT, and NPR Values are represented as percentages. Chi-square results show a significantly higher survival rate associated with NPR and REBOA when compared to EDT (p < 0.001) EDT: emergency department thoracotomy; REBOA: resuscitative endovascular balloon occlusion of the aorta; NPR: nonprocedural resuscitation

EDT patients had a higher mortality rate than REBOA. REBOA, in turn, was associated with higher mortality than NPR. These findings remained significant after removing patients with cardiac arrest. When comparing EDT to REBOA, excluding nonprocedural patients, EDT was still associated with a significantly higher mortality rate. Comparing REBOA and NPR, excluding EDT, showed a significantly lower risk of ED mortality for nonprocedural patients (Table 2, Figure 1). Significance was maintained following the removal of patients with cardiac arrest (Tables 3, 4).

**Table 2:** Comparison of mortality rates in patients presenting with cardiac arrest Chi-square results show no significant difference in mortality between procedural and NPR (p = 0.7188749). Almost all patients who arrived with cardiac arrest did not survive EDT: emergency department thoracotomy; REBOA: resuscitative endovascular balloon occlusion of the aorta; NPR: nonprocedural resuscitation

Procedure	Death		Total (n = 43)
	No (n = 1)	Yes (n = 42)	
EDT	0 (0.000)	31 (1.000)	31 (0.721)
REBOA	0 (0.000)	5 (1.000)	5 (0.116)
NPR	1 (0.143)	6 (0.857)	7 (0.163)

**Table 3:** Comparative analysis of mortality rates in patients presenting without cardiac arrest upon arrival Chi-square results show a significantly higher rate of mortality associated with procedural resuscitation (p < 0.001) EDT: emergency department thoracotomy; REBOA: resuscitative endovascular balloon occlusion of the aorta; NPR: nonprocedural resuscitation

Procedure	Death		Total (n = 224)
	No (n = 151)	Yes (n = 73)	
EDT	7 (0.175)	33 (0.825)	40 (0.179)
REBOA	7 (0.583)	5 (0.417)	12 (0.054)
NPR	137 (0.797)	35 (0.203)	172 (0.768)

Multivariate analysis

**Table 4:** Odds of death adjusted for procedure, severity, body region, demographics, and MOI Odds of death adjusted for type of resuscitation and common indicators of injury severity. Multivariate logistic regression was performed with severity scores, body region, patient demographics, and MOI serving as covariates. The reference group was EDT. Results show significantly higher odds of death associated with EDT when compared to REBOA and even higher association when comparing EDT to NPR. Statistical significance codes: 0.001^*^; 0^**^ REBOA: resuscitative endovascular balloon occlusion of the aorta; NPR: nonprocedural resuscitation; MOI: mechanism of injury; EDT: emergency department thoracotomy

Procedure	Odds ratio	Standard error	z value	p
REBOA	0.09	0.91	-2.66	0.008^*^
NPR	0.02	0.73	-6.18	<0.001^**^

The adjusted odds ratio (OR) for mortality associated with REBOA compared to EDT was 0.09, while the OR for patients who received NPR compared to EDT was 0.02. This means that the odds of death are 10-fold higher in patients who received EDT than in those who received REBOA and 50-fold higher in patients who received EDT than in those who received NPR (Table 4).

Of the 64 EDT patients who died, 58 (90.6%) did not survive acute resuscitation efforts. The remaining six patients (9.4%) achieved hemodynamic stability following EDT but later died during inpatient admission as a result of trauma-related complications. These patients survived an average of 12 days in the hospital with a range of 3-25 days. Complications experienced during hospital admission included anoxic brain injury with cerebral swelling, shock liver and bowel, kidney failure, and acute respiratory distress syndrome. Of the 10 REBOA patients who died, seven patients (70.0%) did not survive initial resuscitation, whereas three patients (3.0%) survived to inpatient admission. Two patients died within four days of the initial trauma, whereas one patient died 38 days after trauma as a result of complications related to wound dehiscence and evisceration. Other in-hospital complications included compartment syndrome and acute kidney injury (AKI), neither of which resulted in patient death.

## Discussion

Our findings show better survival outcomes in patients receiving REBOA compared to EDT, supporting our initial hypothesis. Several meta-analyses and original studies comparing REBOA to EDT have yielded similar results [6,17-22]. However, our results also demonstrate that receiving either invasive intervention, such as REBOA or EDT, was associated with a higher mortality rate compared to NPR. Therefore, REBOA cannot be said to offer survival benefits compared to NPR. One interpretation is that EDT and REBOA are high-risk interventions that could potentially cause life-threatening complications. Both interventions occlude the aorta, thus risking downstream end-organ ischemia and irreversible damage. Complications of EDT include anoxic brain injury, ischemia-reperfusion injury, and damage to surrounding structures resulting in bleeding. Direct complications of the intervention include bleeding from internal mammary arteries following thoracotomy, myocardial or coronary artery damage during pericardiotomy, and esophageal or phrenic nerve damage from cross clamping the aorta [23,24]. REBOA has been associated with increased rates of AKI and lower limb amputations compared to patients who were managed noninvasively [25]. Additional complications include arterial damage when obtaining femoral access and aortoiliac damage with balloon inflation that results in intimal tears, dissection, thrombosis, pseudoaneurysms, thromboemboli, ischemia due to prolonged occlusion, and vessel rupture [13,26,27].

However, despite the complications associated with REBOA and EDT, we theorize that it is unlikely that these procedures are directly responsible for the increased mortality. It is more likely the effect of bias by indication. Bias by indication refers to differences in outcome due to the reasons for treatment rather than the treatment itself [28]. Patients who deteriorated to the point of requiring a salvage maneuver unsurprisingly had worse outcomes than patients who met inclusion criteria but responded to less aggressive treatment. A recent study of REBOA using the National Trauma Data Bank found a harmful association with REBOA that could only be nullified by a potent confounder [29]. We suggest there are indeed important biases that cannot consistently be accounted for in a trauma setting, primarily those contributing to patient acuity and procedure choice, that subsequently impact the relationship between REBOA and negative outcomes. There are likely many additional confounding variables associated with procedure selection, such as clinical gestalt, trends of vitals, or patient response to previous interventions, that we were not able to capture from a registry or chart review. For this reason, these interventions are generally reserved for patients in extremis. These patients are near death but still demonstrate some signs of life, including, but not limited to, pupillary response, spontaneous ventilation, measurable or palpable blood pressure, extremity movement, or cardiac electrical activity.

Notably, the 2023 UK-REBOA trial, representing the first randomized control trial to examine the clinical effectiveness of REBOA, supported the use of REBOA as an effective means of temporary stabilization in patients presenting with NCTH. However, this trial redemonstrated several significant challenges, including complications such as ischemic injury and the lack of long-term survival benefit when compared to standard care, including airway protection, fluid resuscitation, and non-REBOA operative intervention as indicated. This study underscored the need for proper patient selection and timing for REBOA deployment to optimize its benefits [27]. The indications for these salvage procedures are still not entirely clear, and the guidelines continue to lack standardization. For example, a systematic review exploring existing REBOA algorithms found a broad consensus on its use for blunt and penetrating trauma patients with NCTH refractory to blood product resuscitation, as employed in this study. However, algorithms diverge on precise systolic blood pressure triggers for REBOA deployment as well as its use for traumatic arrest and chest or extremity hemorrhage control. Furthermore, many algorithms do not comment on the specific method of identifying major chest or thoracic vascular trauma before a patient selection for REBOA [30]. Similarly, the approach to patient selection for EDT is largely based on the presence or absence of several predictors of survival, such as injury mechanism, anatomic injury location, and degree of physiologic derangement. However, most prior reports have focused on a single survival predictor at a time, making interpretation and extrapolation to clinical practice with complex patients difficult [5]. Future studies should aim to determine whether there are consistently associated parameters that may be used to create universal, evidence-based guidelines outlining when to use REBOA, EDT, or NPR.

Limitations

REBOA and EDT are not always performed for the same indications. REBOA is contraindicated for chest injuries that require EDT in traumatic arrest. This study was limited by a small population, which complicates the ability to draw reliable conclusions and increases the risk of results arising from random variation. As previously discussed, the comparison between EDT and REBOA is confounded by indication. The only way to eliminate this bias would be a randomized controlled trial, which would be difficult given the low numbers. Additionally, given the inconsistency and incompleteness of the trauma documentation, we did not have accurate fluid measurements to report. Fluid resuscitation was performed until the patient was hemodynamically stable or until procedural intervention was indicated. However, it was not feasible to quantify what could represent a potential confounder.

## Conclusions

The association of REBOA with death is lower when compared to EDT, but higher when compared to NPR. REBOA can be considered as a less invasive alternative in the setting of unstable life-threatening abdominopelvic hemorrhage without a therapeutic target in the chest. Given the worse survival compared with NPR we cannot recommend liberalizing its use to patients who can be stabilized with hemostatic volume resuscitation.
